# Role of Interprofessional primary care teams in preventing avoidable hospitalizations and hospital readmissions in Ontario, Canada: a retrospective cohort study

**DOI:** 10.1186/s12913-020-05658-9

**Published:** 2020-08-24

**Authors:** Wissam Haj-Ali, Rahim Moineddin, Brian Hutchison, Walter P. Wodchis, Richard H. Glazier

**Affiliations:** 1 Dalla Lana School of Public Health, Toronto, Ontario Canada; 2 Institute of Health Policy, Management and Evaluation, University of Toronto, 155 College Street, Toronto, Ontario M5T 3M6 Canada; 3 Canadian Centre for Health Economics, Toronto, Canada; 4 Institute for Clinical Evaluative Sciences, Toronto, Canada; 5 Department of Family and Community Medicine, University of Toronto, Toronto, Ontario Canada; 6 Departments of Family Medicine and Health Research Methods, Evidence and Impact, McMaster University, Hamilton, Canada; 7 Trillium Health Partners, Institute for Better Health, Toronto, Ontario Canada; 8 MAP Centre for Urban Health Solutions, St. Michael’s Hospital, Toronto, Canada

**Keywords:** Primary care reform, Primary health care, Avoidable hospitalizations, Health services delivery, Ontario, Canada

## Abstract

**Background:**

Improving health system value and efficiency are considered major policy priorities internationally. Ontario has undergone a primary care reform that included introduction of interprofessional teams. The purpose of this study was to investigate the relationship between receiving care from interprofessional versus non-interprofessional primary care teams and ambulatory care sensitive condition (ACSC) hospitalizations and hospital readmissions.

**Methods:**

Population-based administrative databases were linked to form data extractions of interest between the years of 2003–2005 and 2015–2017 in Ontario, Canada. The data sources were available through ICES. The study design was a retrospective longitudinal cohort. We used a “difference-in-differences” approach for evaluating changes in ACSC hospitalizations and hospital readmissions before and after the introduction of interprofessional team-based primary care while adjusting for physician group, physician and patient characteristics.

**Results:**

As of March 31st, 2017, there were a total of 778 physician groups, of which 465 were blended capitation Family Health Organization (FHOs); 177 FHOs (22.8%) were also interprofessional teams and 288 (37%) were more conventional group practices (“non-interprofessional teams”). In this period, there were a total of 13,480 primary care physicians in Ontario of whom 4848 (36%) were affiliated with FHOs—2311 (17.1%) practicing in interprofessional teams and 2537 (18.8%) practicing in non-interprofessional teams. During that same period, there were 475,611 and 618,363 multi-morbid patients in interprofessional teams and non-interprofessional teams respectively out of a total of 2,920,990 multi-morbid adult patients in Ontario. There was no difference in change over time in ACSC admissions between interprofessional and non-interprofessional teams between the pre- and post intervention periods. There were no statistically significant changes in all cause hospital readmission s between the post- and pre-intervention periods for interprofessional and non-interprofessional teams.

**Conclusions:**

Our study findings indicate that the introduction of interprofessional team-based primary care was not associated with changes in ACSC hospitalization or hospital readmissions. The findings point for the need to couple interprofessional team-based care with other enablers of a strong primary care system to improve health services utilization efficiency.

## Background

Improving health system value and efficiency are considered major policy priorities internationally [1, 2]. While health system costs continue to be a challenge across jurisdictions, hospitalizations for ambulatory care sensitive conditions (ACSCs) and hospital readmissions have been a focus for policymakers [3–6]. ACSC hospitalizations are potentially avoidable by preventing the inception of disease, controlling an acute episodic illness, or managing a chronic condition effectively [7]. When care is delivered to patients when and where they need it, hospital readmissions can sometimes be prevented [8]. Evidence has suggested a link between the burden of multi-morbidity and health services use, particularly hospitalizations [9–12]. Hence, multi-morbid patients continue to be a key focus from a clinical care and population health perspective [13–16]. Interprofessional team-based care may have an important role to play in caring for multi-morbid patients by offering a collaborative approach to prevent ACSC hospitalization and hospital readmissions.

During the 1990s, federal and provincial governments in Canada faced fiscal challenges that resulted in limited healthcare spending and investments in primary care innovation [17]. In the 2000s, Ontario introduced primary care reform in response to the recommendations of various federal and provincial reports [18, 19]. Primary care reform movement in Ontario included three major policy initiatives: new physicians’ reimbursement and organizational models, patient enrolment with a primary care provider and support to interprofessional team-based care [20]. During the last 20 years, more than one third of Ontario primary care physicians have voluntarily transitioned from traditional fee-for-service practice to blended capitation payment and in some cases received additional funding to support interprofessional team members to join their practice [21]. Ontario interprofessional Family Health Teams have many similarities with Quebec Family Medicine Groups, Alberta Primary Care Networks and the Patient-Centered Medical Home in the United States (US) [20, 22–24].

In Ontario, reducing hospitalization for ACSC conditions and all-cause readmission are strategic priorities [6, 25]. In this study, we examined the association between the introduction of primary care interprofessional teams and unplanned ACSC hospital admissions and all cause hospital readmissions among multi-morbid patients. We compared changes in those outcomes over time among physicians remunerated through the same physician payment model, some of whom transitioned to interprofessional team-based practice. We hypothesised that multi-morbid patients who receive care from an interprofessional teams will have lower ACSC hospital admissions and all-cause readmissions over time when compared to patients receiving care from non-interprofessional teams.

## Methods

### Setting

Ontario is the most populous province in Canada with a population of 14.4 million people in 2019 [26]. During the last two decades Ontario primary care services payment and organization have been subject to significant changes. In the early 2000s, primary care physicians were mainly paid on a fee-for-service basis and worked individually. Currently, most primary care physicians work in organised models and are largely paid through capitation. The three dominant practice models in Ontario are: enhanced fee-for-service (85% fee-for-service, 15% capitation and bonuses, no funding for non-physician health professionals); non-interprofessional team blended capitation (20% fee-for-service, 80% capitation and bonuses, no funding for non-physician health professionals), and interprofessional team blended capitation (20% fee-for-service, 80% capitation and bonuses, and funding for non-physician health professionals) [27]. The dominant model in Ontario is Family Health Organization (FHO). Within FHOs groups of physicians can be practicing in either interprofessional or non-interprofessional teams. At minimum, three physician practice together in a FHO to offer comprehensive care. FHOs were eligible to apply for additional funding to become interprofessional teams and typically include primary care physicians and nurses or nurse practitioners and at least one allied health care professional such as pharmacist, social worker or dietitian. Interprofessional teams are also eligible for funding an administrator or executive director and electronic medical records.

### Study design and population

We conducted a retrospective cohort study with longitudinal design given the importance of temporal effect on interprofessional teams formation and maturation and their relationship to the outcomes under investigation. We used the “difference in differences” approach, an econometric method for evaluating changes in outcomes after policy implementation. The difference-in-differences study design compares outcomes after and before the intervention between the study group without the exposure (group A: patients in non-interprofessional teams) and the study group with the exposure (group B: patients in interprofessional teams). Two differences in outcomes are important: the difference after vs before the implementation of interprofessional teams in the group exposed (B2 − B1) and the difference after vs before the implementation of interprofessional teams in the unexposed group (A2 − A1). The change in outcomes that are related to implementation of interprofessional teams beyond background trends can then be estimated from the difference-in-differences analysis as follows: (B2 − B1) − (A2 − A1). If there is no relationship between implementation of interprofessional teams and subsequent outcomes, then the difference-in-differences estimate is equal to 0. In contrast, if the implementation of interprofessional teams is associated with beneficial changes, then the outcomes following implementation will improve in the exposed group [28].

Several population-based administrative databases were linked using unique encoded identifiers at ICES (formerly known as the Institute for Clinical Evaluative Sciences) to form data extractions of interest. We generated a cohort that included the same patients at two different points in time, pre- and post-teams’ formation. The study population included patients between 18 and 105 years old, who had two or more of a list of 17 chronic conditions as identified at the beginning of the pre-teams’ formation period, March 31st 2003 and who were part of a FHO blended capitation model as identified at the beginning of the post-teams formation period, March 31st, 2015. The chronic condition selection was based on clinical relevance and impact on the outcomes being investigated as described in previous literature [29–34]. These conditions have been adopted in previous studies [35, 36] and are consistent with the parameters outlined by the Department of Health and Human Services for defining and measuring chronic conditions [37]. The conditions include: cancer, diabetes, asthma, chronic obstructive pulmonary disease (COPD), hypertension, chronic coronary syndrome (CCS), cardiac arrhythmia, congestive heart failure (CHF), stroke, acute myocardial infarction (AMI), renal failure, arthritis (excluding rheumatoid arthritis), rheumatoid arthritis, osteoporosis, depression, dementia and mental health conditions (full list of diagnostic information for defining the 17 selected chronic conditions under investigation in this study are included in Appendix 1).

The baseline study population included people identified before interprofessional teams formation who were still identifiable after interprofessional teams formation and were part of the FHO blended capitation model. People in the baseline population were followed-up to February 28th, 2005 for first unplanned ACSC admission and up to March 31st, 2005 for first all-cause readmission and in the follow up period up to February 28th, 2017 for the first ACSC admission and up to March 31st, 2017 for all-cause readmission. Given that teams did not exist during the baseline period, assignment of patients to interprofessional and non-interprofessional teams was based on their post-intervention assignment. We excluded individuals who died and individuals who were in long term care or complex continuing care.

### Measures and data sources

#### ACSC admission and hospital readmission

The primary outcome was hospital admissions for ACSCs, defined as the first hospital non-elective admission with a most responsible diagnosis code of: grand mal status and other epileptic convulsions, chronic obstructive pulmonary disease (COPD), asthma, diabetes, heart failure and pulmonary edema, hypertension and angina.

The secondary outcome was hospital readmissions, defined as the first subsequent non-elective all-cause readmission to an acute care hospital within 30 days of discharge, among hospitalisation for selected Case Mix Group (CMG) groups: stroke, COPD, pneumonia, congestive heart failure, diabetes, cardiac conditions, gastrointestinal conditions (List of CMGs codes in Appendix 2). The primary and secondary outcomes were derived from the OHIP database and the Discharge Abstract Database (DAD) and the Registered Patient Database (RPDB) available at ICES. Both outcomes excluded people without a valid date of admission/discharge; and people who died during their hospital stay (relevant to admission but not readmission).

#### Physician group and physicians characteristics

Physician group and physician characteristics were derived from a health care provider data registry available at ICES. Physician group characteristics included the number of physicians per group and number of years under the capitation model. Physicians’ characteristics included age, sex, Canadian graduate status and number of years in practice.

#### Patient characteristics

Patient characteristics were identified from a population and demographics data registry available at ICES. Patients’ characteristics included age, sex and recent OHIP registration as a proxy for immigration (might include recent registrants that moved from other provinces). By linking patients’ postal code to census data we were able to derive neighborhood income quintiles—quintile 1 having the lowest relative income and quintile 5 the highest. The Ontario Medical Association Rurality Index of Ontario (RIO) was used to identify rurality with a score ranging from zero (most urban) to 100 (most rural) [38].

The Resource Utilization Bands (RUBs) categories ranging from 0 (no expected utilization) to 5 (very high expected utilization) were based on the Johns Hopkins Adjusted Clinical Groups case-mix system software [39].

Six chronic diseases conditions (AMI, asthma, CHF, COPD, hypertension, diabetes) were defined based on previously validated population-derived ICES cohorts [40–45]. For the conditions where a derived ICES cohort was not available (cancer, cardiac arrhythmia, chronic coronary syndrome, dementia, depression, arthritis (excluding rheumatoid arthritis), osteoporosis, renal failure, rheumatoid arthritis, and stroke), a similar approach for the derivation was adopted—at least one diagnosis recorded in acute care, or two diagnoses recorded in physicians’ records within a two-year period. The conditions were derived using the DAD and OHIP databases available at ICES.

### Statistical analysis

For the descriptive results, we generated frequencies, percentages, means and standard deviations to describe the characteristics of physician groups, physicians and patients who are either in interprofessional teams or non-teams and their respective admission and readmission rates.

For the admission and readmission models, as a first step we tested for patient clustering within physicians using a random effects logistics regression. Clustering was not significant. As a result, we ran ordinary logistic regression models with binary outcomes of ACSC admission and all-cause readmission. The independent variables added to the models were the respective physician group, physician and patient characteristics.

To estimate the difference in differences we used Generalized Estimating Equations method to account for repeated measures within patients. The independent variables added to the models were the respective physician group, physician and patient characteristics.

All study analyses were conducted using SAS v.9.3 and statistical significance was assessed at a *p*-value < 0.05.

### Ethics approval

The use of data in this project was authorized under section 45 of Ontario’s Personal Health Information Protection Act, which does not require review by a research ethics board.

## Results

### Baseline physician group, physician and patient characteristics comparing interprofessional teams to non-interprofessional teams

As of March 31st^,^ 2017, there were a total of 778 physician groups in Ontario, of which 465 were FHOs; 177 FHOs (22.8%) were also interprofessional teams and 288 (37%) were non-interprofessional teams. Compared to non-interprofessional teams, interprofessional teams had: more physicians per group and more years under the capitation model.

In this period, there were a total of 13,480 primary care physicians in Ontario of whom 4848 (36%) were affiliated with FHOs, 2311 (17.1%) practicing in interprofessional teams and 2537 (18.8%) practicing in non-interprofessional teams. Compared to non-interprofessional teams, interprofessional teams had fewer patients per physician, more female physicians, more physicians in the younger age group, more physicians who were Canadian graduates and fewer years in practice (Table 1).

**Table 1:** Physicians group and physicians characteristics by enrolment model of care – comparing interprofessional teams to non-interprofessional teams to all groups (patient enrolment models) in Ontario based on March 31st, 2015

	Interprofessional Teams		Non-interprofessional teams		All Ontario physician groups (patient enrolment models) and physicians	
**Physicians’ Group characteristics**						
Groups No. (% of all PEMs)	177	22.8	288	37.0	778	100.0
Number of physicians per group, Mean (SD)	13.11	10.7	8.8	7.6	17	188.9
Years under the capitation model, Mean (SD)	6.00	3.0	4.3	2.6	6	3.3
**Physicians characteristics**						
**Physicians No. (% of all physicians)**	2311	17.1	2537	18.8	13,480	100.0
Number of patients per physician, Mean (SD)	1303	638.9	1517	675.9	1020	944.6
**Sex No. (%)**						
Male	1212	52.4	1391	54.8	7270	53.9
Female	1099	47.6	1146	45.2	5864	43.5
Missing	0	0.0	0	0.0	346	2.6
**Age group No. (%) in Yrs.**						
< 40	546	23.6	364	14.4	2518	18.7
40–64	1499	64.9	1773	69.9	7930	58.8
> 64	232	10.0	373	14.7	2031	15.1
Missing	34	1.5	27	1.1	1001	7.4
**Country of medical graduation Canada No. (%)**						
Yes	1874	81.1	1871	73.8	8974	66.6
No	403	17.4	639	25.2	3505	26.0
Missing	34	1.5	27	1.1	1001	7.4
**Years in practice No. (%)**						
< 5	60	2.6	48	1.9	667	5.0
5_15	701	30.3	465	18.3	3145	23.3
16–25	531	23.0	645	25.4	3047	22.6
> 25	1019	44.1	1379	54.4	6275	46.6
Missing	0	0.0	0	0.0	346	2.6

During the same period, there were 475,611 and 618,363 multi-morbid patients in interprofessional and non-interprofessional teams respectively out of a total of 2,920,990 multi-morbid adult patients in Ontario. Overall interprofessional teams had fewer new immigrant patients and more patients who reside in rural areas. Other patient characteristics were relatively similar between interprofessional and non-interprofessional teams. When compared to all physician groups, both interprofessional and non-interprofessional teams had less patients with high number of co-morbidities (Table 2).

**Table 2:** Patients’ characteristics comparing patients in interprofessional teams, non-interprofessional teams, all multi-morbid patients and all Ontarians adults on March 31st, 2003

	Multi-morbid patients in interprofessional teams		Multi-morbid patients in Non- interprofessional teams		All multi-morbid patients in Ontario		All Ontarians	
Patients total	475,611		618,363		2,920,990		9,397,586	
**Sex No. (%)**								
Males	186,729	39.3	246,882	39.9	1,240,516	42.5	4,576,936	48.7
Female	288,882	60.7	371,481	60.1	1,680,474	57.5	4,820,650	51.3
Missing	–	0.0	–	0.0	–	0.0	–	0.0
**Age group, yr. No. (%)**								
18–44	138,965	29.2	184,059	29.8	654,813	22.4	4,863,276	51.8
45–64	227,930	47.9	296,914	48.0	1,127,265	38.6	2,981,705	31.7
65–84	107,821	22.7	136,227	22.0	999,353	34.2	1,389,782	14.8
84+	895	0.2	1163	0.2	139,559	4.8	162,823	1.7
Missing	–	0.0	–	0.0	–	0.0	–	0.0
**New OHIP registrants (within 10 years) No. (%)**	13,742	2.9	29,981	4.9	157,488	5.4	1,200,951	12.8
**Income quintile, No. (%)**								
1 (low)	84,198	17.7	101,739	16.5	583,685	20.0	1,799,279	19.2
2	96,387	20.3	115,903	18.7	605,293	20.7	1,884,459	20.1
3	95,925	20.2	125,618	20.3	588,141	20.1	1,892,274	20.1
4	96,214	20.2	132,243	21.4	570,140	19.5	1,903,560	20.3
5 (high)	101,596	21.4	141,926	23.0	565,536	19.4	1,888,811	20.1
Missing	1291	0.3	934	0.2	8195	0.3	29,203	0.3
**Rurality Index of Ontario, No. (%)**								
Major urban (0 to 9)	257,792	54.2	475,286	76.9	2,026,660	69.4	6698,329	71.3
Semi-urban (10 to 39)	150,810	31.7	111,986	18.1	608,960	20.9	1,852,225	19.7
Rural (≥40)	63,866	13.4	28,970	4.7	260,936	8.9	761,861	8.1
Missing	3143	0.7	2121	0.3	24,434	0.8	85,171	0.9
**Resource utilization band (RUB), No. (%)**								
0 (non-user)	2157	0.5	2431	0.4	30,338	1.0	938,240	10.0
1	2252	0.5	2595	0.4	11,227	0.4	555,466	5.9
2	23,325	4.9	27,403	4.4	114,781	3.9	1,588,712	16.9
3	306,213	64.4	399,620	64.6	1,691,226	57.9	4,685,817	49.9
4	109,010	22.9	146,389	23.7	734,298	25.1	1,253,298	13.3
5 (very high user)	32,654	6.9	39,925	6.5	339,120	11.6	376,053	4.0
Missing								
**Patients with Chronic disease**								
2 + Co-morbidity No. (%)	475,611	100.0	618,363	100.0	2,920,990	100.0	2,920,990	31.1
3+ comorbidities No. (%)	194,828	41.0	257,141	41.6	1,481,098	50.7	1,481,098	15.8
4+ comorbidities No. (%)	71,285	15.0	95,323	15.4	723,296	24.8	723,296	7.7
5+ comorbidities No. (%)	23,824	5.0	323,368	5.2	344,685	11.8	344,685	3.7

### ACSC hospital admissions and all cause 30-day re-admissions in interprofessional teams and non-interprofessional teams by physician and patient characteristics

During the period of April 1st, 2015 to March 31st, 2017, the unadjusted results showed that interprofessional teams were found to have higher ACSC admission rates when compared to non-interprofessional teams (2.5% versus 2.1%, respectively). When we stratified the results by physician characteristics, the following had a higher ACSC admission rate: males, older physicians, and non-Canadian graduates (Table 3). When we stratified the results by patient characteristics, the following had a higher ACSC admission rate: males, older patients, non-immigrants, patients in the lowest neighborhood income quintile, residents of a rural area, patients in the highest expected resource utilization band and patients with five and plus co-morbidities (Table 4).

**Table 3:** ACSC hospital admissions between April 1st, 2015 and February 28th, 2017 among multi-morbid adults by physician characteristics identified on March 31st, 2015

	Interprofessional Teams			Non-interprofessional teams			
	Numerator	Denominator	Rate per 100	Numerator	Denominator	Rate per 100	Rate Difference (interprofessional Teams - Non-interprofessional teams)
**ACSC admissions and patients totals**	**11,963**	**475,611**	**2.5**	**13,160**	**618,363**	**2.1**	**0.4**
**Physicians characteristics**							
**Sex**							
Male	8183	298,763	2.7	9547	407,328	2.3	0.4
Female	3780	176,848	2.1	3613	210,599	1.7	0.4
Missing					436	0.0	
**Age group**							
< 40	2013	80,487	2.5	1098	54,012	2.0	0.5
40–64	8170	332,177	2.5	9242	445,990	2.1	0.4
> 64	1648	58,240	2.8	2730	114,424	2.4	0.4
Missing	132	4707	2.8	90	3937	2.3	0.5
**Country of medical graduation Canada**							
Yes	9389	379,843	2.5	9459	456,855	2.1	0.4
No	2442	91,061	2.7	3611	157,571	2.3	0.4
Missing	132	4707	2.8	90	3937	2.3	0.5
**Years in practice**							
< 5	246	9457	2.6	180	6971	2.6	0.0
5_15	2650	105,104	2.5	1464	71,094	2.1	0.4
16–25	2571	107,080	2.4	3047	144,860	2.1	0.3
> 25	6496	253,970	2.6	8460	395,002	2.1	0.5
Missing		–		9	436	2.1	−2.1

**Table 4:** ACSC hospital admissions between April 1st, 2015 and March31st, 2017 among multi-morbid adults by patient characteristics from March 31st, 2003

	Patients characteristics						
	Numerator	Denominator	Rate per 100				
ACSC admissions and patients totals	11,963	475,611	2.52	13,160	618,363	2.13	0.39
**Sex**							
Males	5265	186,729	2.8	5869	246,882	2.4	0.4
Female	6698	288,882	2.3	7291	371,481	2.0	0.3
Missing	–	–		–	–	0.0	
**Age group, yr.**							
18–44	1229	138,965	0.9	1288	184,059	0.7	0.2
45–64	5213	227,930	2.3	5665	296,914	1.9	0.4
65+	5521	108,716	5.1	6207	137,390	4.5	0.6
Missing		–			–	0.0	
**New OHIP registrants (within 10 years)**							
Yes	294	13,742	2.1	470	29,981	1.6	0.5
No	11,669	461,869	2.5	12,690	588,382	2.2	0.3
**Income quintile**							
1 (low)	2742	84,198	3.3	2859	101,739	2.8	0.5
2	2710	96,387	2.8	2815	115,903	2.4	0.4
3	2338	95,925	2.4	2631	125,618	2.1	0.3
4	2161	96,214	2.2	2545	132,243	1.9	0.3
5 (high)	1972	101,596	1.9	2290	141,926	1.6	0.3
Missing	40	1291	3.1	20	934	2.1	1
**Rurality Index of Ontario**							
Major urban (0 to 9)	5741	257,792	2.2	9396	475,286	2.0	0.2
Semi-urban (10 to 39)	4062	150,810	2.7	2809	111,986	2.5	0.2
Rural (≥40)	2060	63,866	3.2	881	28,970	3.0	0.2
Missing	100	3143	3.2	74	2121	3.5	−0.3
**Resource utilization band (RUB)**							
0 (non-user)	37	2157	1.7	56	2431	2.3	−0.6
1	40	2252	1.8	27	2595	1.0	0.8
2	399	23,325	1.7	382	27,403	1.4	0.3
3	6410	306,213	2.1	7081	399,620	1.8	0.3
4	3370	109,010	3.1	3773	146,389	2.6	0.5
5 (very high user)	1707	32,654	5.2	1841	39,925	4.6	0.6
Missing							
**Patients with Chronic disease**							
2 + Co-morbidity							
Yes	11,963	475,611	2.5	13,160	618,363	2.1	0.4
No	–	–		–	–		
3+ comorbidities							
Yes	7635	257,141	3.0	8657	257,141	3.4	−0.4
No	4328	280,783	1.5	4503	361,222	1.2	0.3
4+ comorbidities							
Yes	4213	71,285	5.9	4841	95,323	5.1	0.8
No	7750	404,326	1.9	8319	523,040	1.6	0.3
5+ comorbidities							
Yes	1949	23,824	8.2	2329	32,368	7.2	1
No	10,014	451,787	2.2	10,831	585,995	1.8	0.4

During that same period, the unadjusted results showed that interprofessional teams had a slightly higher all cause hospital 30-day re-admission rate when compared to non-interprofessional teams (15.0% versus 14.6%, respectively). When we stratified the results by physician characteristics, we found that non-Canadian graduates had a higher readmission rate (Table 5). When we stratified the results by patient characteristics, the following had a higher readmission rate: males, patients in the older age category, residents of major urban areas, patients in the highest expected resource utilization band and patients with five or more co-morbidities (Table 6).

**Table 5:** All cause hospital readmissions among multi-morbid adults between April 1^st^, 2015 and March 31st, 2017 by physician characteristics based March 31st, 2017

	Interprofessional Teams			Non-interprofessional teams			
	Numerator	Denominator	Rate per 100	Numerator	Denominator	Rate per 100	Rate Difference (Interprofessional Teams - Non-interprofessional teams)
**All-cause readmissions and patient totals**	**1796**	**11,963**	**15.0**	**1917**	**13,160**	**14.6**	**0.4**
**Sex No. (%)**							
Male	**1231**	**8183**	**15.0**	**1375**	**9547.00**	**14.4**	**0.6**
Female	**565**	**3780**	**14.9**	**542**	**3613.00**	**15.0**	**−0.1**
Missing	**0**	**0**	**0.0**	**0**	**0.00**	**0.0**	**0**
**Age group No. (%) in Yrs.**							
< 40	**320**	**2013**	**15.9**	**156**	**1098.00**	**14.2**	**1.7**
40–64	**1208**	**8170**	**14.8**	**1346**	**9242.00**	**14.6**	**0.2**
65+	**255**	**1648**	**15.5**	**404**	**2730.00**	**14.8**	**0.7**
Missing	**13**	**132**	**9.8**	**11**	**90.00**	**12.2**	**−2.4**
**Country of medical graduation Canada No. (%)**							
Yes	**1405**	**9389**	**15.0**	**1369**	**9459.00**	**14.5**	**0.5**
No	**378**	**2442**	**15.5**	**537**	**3611.00**	**14.9**	**0.6**
Missing	**13**	**132**	**9.8**	**11**	**90.00**	**12.2**	**−2.4**
**Years in practice No. (%)**							
< 5	**36**	**246**	**14.6**	**24**	**189.00**	**12.7**	**1.9**
5_15	**406**	**2650**	**15.3**	**204**	**1464.00**	**13.9**	**1.4**
16–25	**385**	**2571**	**15.0**	**437**	**3047.00**	**14.3**	**0.7**
> 25	**969**	**6496**	**14.9**	**1252**	**8460.00**	**14.8**	**0.1**
Missing	**0**	**0**	**0.0**	**0**	**0.00**	**0.00**	**0**

**Table 6:** All cause hospital readmissions between April 1st, 2015 and March31st, 2017 among multi-morbid adults by patient characteristics from March 31st, 2003

Patients characteristics							
**All cause readmission s and patient totals**	**1796**	**11,963**	**15.0**	**1917**	**13,160**	**14.6**	**0.4**
**Sex No. (%)**							
Males	**807**	**5265**	**15.3**	**893**	**5869**	**15.2**	**0.1**
Female	**989**	**6698**	**14.8**	**1024**	**7291**	**14.0**	**0.8**
Missing		**–**			**–**		**0**
**Age group, yr. No. (%)**							
18–44	**159**	**1229**	**12.9**	**156**	**1288**	**12.1**	**0.8**
45–64	**774**	**5213**	**14.8**	**787**	**5665**	**13.9**	**0.9**
65+	**863**	**5521**	**15.6**	**974**	**6207**	**15.7**	**−0.1**
Missing							
**New OHIP registrants (within 10 years) No. (%)**							
Yes	**36**	**294**	**12.2**	**78**	**470**	**16.6**	**−4.4**
No	**1760**	**11,669**	**15.1**	**1839**	**12,690**	**14.5**	**0.6**
**Income quintile, No. (%)**							
1 (low)	**404**	**2742**	**14.7**	**453**	**2859**	**15.8**	**−1.1**
2	**423**	**2710**	**15.6**	**396**	**2815**	**14.1**	**1.5**
3	**D/S**	**D/S**	**D/S**	**D/S**	**D/S**	**D/S**	**D/S**
4	**349**	**2161**	**16.1**	**360**	**2545**	**14.1**	**2**
5 (high)	**294**	**1972**	**14.9**	**340**	**2290**	**14.8**	**0.1**
Missing	**D/S**	**D/S**	**D/S**	**D/S**	**D/S**	**D/S**	**D/S**
**Rurality Index of Ontario, No. (%)**							
Major urban (0 to 9)	**886**	**5741**	**15.4**	**1403**	**9396**	**14.9**	**0.5**
Semi-urban (10 to 39)	**D/S**	**D/S**	**D/S**	**D/S**	**D/S**	**D/S**	**D/S**
Rural (≥40)	**310**	**2060**	**15.0**	**115**	**881**	**13.1**	**1.9**
Missing	**D/S**	**D/S**	**D/S**	**D/S**	**D/S**	**D/S**	
**Resource utilization band (RUB), No. (%)**							
0 (non-user)	**D/S**	**D/S**	**D/S**	**D/S**	**D/S**	**D/S**	**D/S**
1	**6**	**40**	**15.0**	**7**	**27**	**25.9**	**−10.9**
2	**56**	**399**	**14.0**	**54**	**382**	**14.1**	**−0.1**
3	**D/S**	**D/S**	**D/S**	**D/S**	**D/S**	**D/S**	**D/S**
4	**524**	**3370**	**15.5**	**534**	**3773**	**14.2**	**1.3**
5 (very high user)	**289**	**1707**	**16.9**	**302**	**1841**	**16.4**	**0.5**
Missing							
**Patients with Chronic disease**							
2 + Co-morbidity No. (%)							
yes	**1796**	**11,963**	**15.0**	**1917**	**13,160**	**14.6**	**0.4**
No	**0**	**0**		**0**	**–**		**0**
3+ comorbidities No. (%)							
yes	**1226**	**7635**	**16.1**	**1335**	**8657**	**15.4**	**0.7**
No	**570**	**4328**	**13.2**	**582**	**4503**	**12.9**	**0.3**
4+ comorbidities No. (%)							
yes	**697**	**4213**	**16.5**	**770**	**4841**	**15.9**	**0.6**
No	**1099**	**7750**	**14.2**	**1147**	**8319**	**13.8**	**0.4**
5+ comorbidities No. (%)							
yes	**344**	**1949**	**17.7**	**378**	**2329**	**16.2**	**1.5**
No	**1452**	**10,014**	**14.5**	**1539**	**10,831**	**14.2**	**0.3**

D/S refers to data supressed for observations with a count between 1 and 5 and have been suppressed to comply with Personal Health Information Protection Act privacy legislation

When we stratified the results by males and females for both outcomes, we did not identify sex differences (results not presented but can be made available on request).

### Association between enrolment in an interprofessional team model and ACSC hospital admission and all cause hospital readmission

During the post-intervention period, when we adjusted for physician group, physician and patient characteristics, being in an interprofessional team increased the likelihood of having ACSC hospital admission by 7%. For the same period, we did not find significant difference between interprofessional and non-interprofessional teams for hospital all cause readmission (Table 7).

**Table 7:** Association between enrolment in an interprofessional team-based model and ACSC admissions and all cause hospital readmissions post intervention April 1^st^, 2015 to March 31st, 2017

	**Interprofessional team ACSC Admissions (Reference: Non-Interprofessional teams)**			
	**OR**	**95% CI**		***P*-Value**
**Unadjusted** (null model)	1.19	1.16	1.22	<.0001
**Adjusted**^a^ for**:**				
Physician group characteristics	1.15	1.12	1.18	<.0001
Group and physician characteristics	1.17	1.13	1.18	<.0001
Group, physician and patients	1.07	1.04	1.18	<.0001
	**Interprofessional team readmission s (Reference: non-teams)**			
	**OR**	**95% CI**		***P*-Value**
**Unadjusted** (null model)	1.31	0.98	1.75	0.073
**Adjusted**^a^ for**:**				
Physician group characteristics	1.17	0.86	1.60	0.323
Group and physician characteristics	1.17	0.84	1.60	0.323
Group, physician and patients	1.20	0.84	1.65	0.260

^a^Adjustment used physician groups and physicians’ characteristics from March 31st, 2015 (post-intervention) and patients’ characteristics from March 31st, 2003 (pre-intervention)

When we examined difference in ACSC hospital admission during the after and before periods the difference was the 1.34% among both interprofessional teams (B2-B1) and non-interprofessional teams (A2-A1). Hence, there was no difference-in-differences (B2 − B1) − (A2 − A1).

When we examined difference in hospital readmission during the after and before periods the difference was 4.90% (*p*-value 0.0003) among interprofessional teams (B2-B1) and 1.47% (*p*-value 0.2798) among non-interprofessional teams (A2-A1). The difference-indifferences (B2 − B1) − (A2 − A1) was non-significant at 3.43% (*p*-value 0.0975) (Table 8).

**Table 8:** Difference in differences model: difference in change over time in ACSC admissions and all cause readmission s between interprofessional teams and non-interprofessional teams from pre-intervention (April 1st, 2003 to March 31st, 2005) to post-intervention (April 1st, 2015 to March 31st, 2017) periods

	Interprofessional Teams						Non- Interprofessional teams							
	2015–17		2003–05		Difference (2015 to 2017–2003 to 2005)		2015–17		2003–05		Difference (2015 to 2017–2003 to 2005)		Difference in differences (diff. Teams – diff. Non-teams)	
**Unplanned ACSC admission**	**Rate per 100**	***P*-value**	**Rate per 100**	***P*-value**	**Rate per 100**	***P*-value**	**Rate per 100**	***P*-value**	**Rate per 100**	***P*-value**	**Rate per 100**	***P*-value**	**Rate per 100**	***P*-value**
Unadjusted model	2.52	<.0001	1.07	<.0001	1.44	<.0001	2.13	<.0001	0.84	<.0001	1.29	<.0001	0.15	0.0008
^a^Adjusted for physician group characteristics	2.48	<.0001	1.06	<.0001	1.42	<.0001	2.15	<.0001	0.85	<.0001	1.30	<.0001	0.12	0.0008
^a^Adjusted for physician group and physician characteristics	2.43	<.0001	1.04	<.0001	1.39	<.0001	2.07	<.0001	0.82	<.0001	1.25	<.0001	0.14	0.0011
^a^Adjusted for physician group and physician and patient characteristics	2.31	<.0001	0.97	<.0001	1.34	<.0001	2.20	<.0001	0.86	<.0001	1.34	<.0001	0.00	0.0016
**Unplanned all cause hospital readmission**	**Rate per 100**	***P*-value**	**Rate per 100**	***P*-value**	**Rate per 100**	***P*-value**	**Rate per 100**	***P*-value**	**Rate per 100**	***P*-value**	**Rate per 100**	***P*-value**	**Rate per 100**	***P*-value**
Unadjusted model	17.71	<.0001	10.90	<.0001	6.81	0.0002	14.26	<.0001	11.96	<.0001	2.30	0.2191	4.51	0.1066
^a^Adjusted for physician group characteristics	17.36	<.0001	10.66	<.0001	6.70	0.0002	14.55	<.0001	12.21	<.0001	2.34	0.219	4.36	0.1062
^a^Adjusted for physician group and physician characteristics	20.30	<.0001	12.73	<.0001	7.57	0.0003	16.76	<.0001	14.39	<.0001	2.37	0.2806	5.20	0.0972
^a^Adjusted for physician group and physician and patient characteristics	12.38	<.0001	7.48	<.0001	4.90	0.0003	9.67	<.0001	8.20	<.0001	1.47	0.2798	3.43	0.0975

^a^Adjustment used physician groups and physicians’ characteristics from March 31st, 2015 (post-intervention) and patients’ characteristics from March 31st, 2003 (pre-intervention)

## Discussion

We used administrative databases to assess the association between receiving care from interprofessional and non-interprofessional primary care teams and unplanned ACSC hospitalizations and all cause hospital readmissions among multi-morbid patients. We followed the same patients before and after teams were implemented which allowed an assessment of the effect of the intervention—introduction of interprofessional team-based care. When we investigated the outcomes during the most recent available period of April 1st, 2015 to March 31st, 2017 interprofessional teams were found to have higher ACSC admission and hospital readmission rates as compared to non-interprofessional teams. However, when we compared the outcomes over time, interprofessional teams were not associated with either an increase or a reduction of ACSC hospital admission and hospital readmission.

The results are consistent with previous evidence that looked at utilization in relation to interprofessional team-based care and found differences in quality but not in healthcare utilization and cost [46–49]. One US study that evaluated the effect of multiplayer patient-centred medical home on healthcare utilization did not find a significant reduction in inpatient admissions [50]. In contrast, several studies from the US assessed multiple components of the medical home model on health services utilization and found significant lower rates of avoidable hospitalization when more medical homeness was incorporated in the health system [51–53]. Implementation of Family Health Teams appeared to contribute to a reduction in ACSC hospitalizations in a Brazilian metropolis, Belo Horizonte [54].

There is a body of evidence that links chronic disease management programs to lower preventable hospitalizations [55–58]. In Ontario, patients being served by both interprofessional and non-interprofessional teams have access to certain chronic disease programs including diabetes education and heart failure clinics. This could be one of the reasons for the absence of difference in our study between receiving care from interprofessional and non-interprofessional teams in ACSC hospitalizations. Additionally, there is heterogeneity of interprofessional teams features across Ontario. Interprofessional team’s composition and the skills mix vary across the different teams. Some interprofessional teams are co-located others are not. Hence, some interprofessional teams might not be ideally set up for care coordination and continuity of care. Continuity of care might be reduced within interprofessional teams if they are not well coordinated and might present a potential for fragmented care. Available evidence from a systematic review suggests that having an accessible and a long-term relationship with a primary care provider appeared to be more important in reducing potentially avoidable hospitalizations than how the primary care delivery is organized. Long-term relationships between primary care physicians and patients reduces hospitalizations for chronic ACSCs and continuity of care has been associated with both reduced health services utilization and patient satisfaction [59–61]. Continuity of care is critical to ensuring that everyone with chronic medical needs receive effective, timely and safe health care [52].

Based on Startfield’s model a strong primary care system should be the first contact for care, as well as continuous, comprehensive and well-coordinated to reduce unwanted outcomes such as preventable hospitalizations [62]. It is important for any jurisdiction that has embarked on or is planning to set up primary care interprofessional team-based care to nurture all these enablers for a strong primary care system.

Our study has several limitations that should be acknowledged. First, administrative databases have not been originally set up for research purposes, which presented a potential for measurement error. However, all the databases used in our study have been validated in Ontario’s context. Additionally, any potential measurement error will be non-deferential between interprofessional and non-interprofessional teams and should not bias the results in a meaningful way. Second, this is an observational study and is susceptible to unmeasured confounding. However, by comparing the outcomes over time, potential risk of bias from unmeasured confounders was limited. Third, due to the adopted study design, to be included in the study population, patients had to survive throughout the study period—April 1st, 2003 to March 31st, 2017. However, a potential survival bias would have affected both interprofessional and non-interprofessional teams’ patients equally and does not present a threat to internal validity. Fourth, ACSC medical admissions and all-cause readmissions are not all unnecessary and preventable. In contrast, in some cases, admission and readmission could be appropriate and reflect appropriate care in the community that flagged the need to be hospitalised.

## Conclusion

Our study findings indicate that the introduction of interprofessional team-based primary care was not associated with reduction in avoidable hospitalizations and hospital readmissions. Those results were not in-line with our hypothesis as we expected that, over time, interprofessional teams would reduce the likelihood of ACSC admissions and readmissions. For jurisdictions aiming to expand physician participation in teams, our study results point to the need to couple interprofessional team-based care with other enablers of a strong primary care system such as access, continuity, comprehensiveness and coordination. Policies and practices that enhance those features will help to implement interprofessional team-based care in a way that it is best able to deliver on intended outcomes such as improving health services utilization efficiency.

## Data Availability

The dataset from this study is held securely in coded form at ICES. While data sharing agreements prohibit ICES from making the dataset publicly available, access may be granted to those who meet pre-specified criteria for confidential access, available at www.ices.on.ca/DAS. The full dataset creation plan and underlying analytic code are available from the authors upon request, understanding that the computer programs may rely upon coding templates or macros that are unique to ICES and are therefore either inaccessible or may require modification.

## Appendix 1

### List of diagnostic information for defining the 17 selected chronic conditions under investigation in this study

**Table 9:** These conditions represent a subset of all possible chronic conditions that may be experienced by individuals over a lifetime but represent the most substantial conditions from a population perspective.

Condition [reference for validated algorithm]	ICD 9 / OHIP	ICD 10	ODB^a^
Acute Myocardial Infarction (AMI) [1]	410	I21, I22	
Osteo- and other Arthritis:			
(A) Osteoarthritis	715	M15-M19	
(B) Other Arthritis (includes Synovitis, Fibrositis, Connective tissue disorders, Ankylosing spondylitis, Gout Traumatic arthritis, pyogenic arthritis, Joint derangement, Dupuytren’s contracture, Other MSK disorders)	727, 729, 710, 720, 274, 716, 711, 718, 728, 739	M00-M03, M07, M10, M11-M14, M20-M25, M30-M36, M65-M79	
Arthritis - Rheumatoid arthritis [2]	714	M05-M06	
Asthma [3]	493	J45	
(all) Cancers	140–239	C00-C26, C30-C44, C45-C97	
Cardiac Arrhythmia	427 (OHIP) / 427.3 (DAD)	I48.0, I48.1	
Congestive Heart Failure [4]	428	I500, I501, I509	
Chronic Obstructive Pulmonary Disease [5]	491, 492, 496	J41, J43, J44	
Coronary syndrome (excluding AMI)	411–414	I20, I22-I25	
Dementia [6]	290, 331 (OHIP) / 046.1, 290.0, 290.1, 290.2, 290.3, 290.4, 294, 331.0, 331.1, 331.5, F331.82 (DAD)	F00, F01, F02, F03, G30	Cholinesterase Inhibitors
Diabetes [7]	250	E08 - E13	
Hypertension [8]	401, 402, 403, 404, 405	I10, I11, I12, I13, I15	
Inflomatary Bowel Disease (IBD) [9]	555, 556	K50, k51	
(Other) Mental Illnesses	291, 292, 295, 297, 298, 299, 301, 302, 303, 304, 305, 306, 307, 313, 314, 315, 319	F04, F050, F058, F059, F060, F061, F062, F063, F064, F07, F08, F10, F11, F12, F13, F14, F15, F16, F17, F18, F19, F20, F21, F22, F23, F24, F25, F26, F27, F28, F29, F340, F35, F36, F37, F430, F439, F453, F454, F458, F46, F47, F49, F50, F51, F52, F531, F538, F539, F54, F55, F56, F57, F58, F59, F60, F61, F62, F63, F64, F65, F66, F67, F681, F688, F69, F70, F71, F72, F73, F74, F75, F76, F77, F78, F79, F80, F81, F82, F83, F84, F85, F86, F87, F88, F89, F90, F91, F92, F931, F932, F933, F938, F939, F94, F95, F96, F97, F98	
Mood, anxiety, depression and other nonpsychotic disorders	296, 300, 309, 311	F30, F31, F32, F33, F34 (excl. F34.0), F38, F39, F40, F41, F42, F43.1, F43.2, F43.8, F44, F45.0, F45.1, F45.2, F48, F53.0, F68.0, F93.0, F99	
Osteoporosis	733	M81, M82	
Renal failure	403, 404, 584, 585, 586, v451	N17, N18, N19, T82.4, Z49.2, Z99.2	
Stroke (excluding transient ischemic attack)	430, 431, 432, 434, 436	I60-I64	

*Abbreviations*: *ICD* International Classification of Disease, *ODB* Ontario Drug Benefit program database, *OHIP* Ontario Health Insurance Plan, physician billings database

All case definitions look back to 2001 to ascertain disease status, with the exception of AMI (1 year prior to index), Cancer (2 years), Mood Disorder (2 years) and Other Mental Illnesses (2 years)

AMI, Asthma, COPD, CHF, Dementia, Diabetes Hypertension and Rheumatoid Arthritis are based on validated case algorithms (see Sources 1–8 below, respectively). All other conditions required at least one diagnosis recorded in acute care (CIHI) or two diagnoses recorded in physician billings within a two-year period

^a^ODB prescription drug records are not available for the majority of persons under the age of 65

## Appendix 2

**Table 10:** List of Eligible CMGs for hospital readmission

List of Eligible Conditions (CMGs)		
CMG+		CMG+ description
**Stroke (Age ≥ 45)**		
**CMG 2008**	25	Hemorrhagic Event of Central Nervous System
	26	Ischemic Event of Central Nervous System
	28	Unspecified Stroke
**CMG 2009**	25	Hemorrhagic Event of Central Nervous System
	26	Ischemic Event of Central Nervous System
	28	Unspecified Stroke
**COPD (Age ≥ 45)**		
**CMG 2008**	139	Chronic Obstructive Pulmonary Disease
**CMG 2009**	139	Chronic Obstructive Pulmonary Disease
**Pneumonia (All ages)**		
**CMG 2008**	136	Bacterial Pneumonia
	138	Viral/Unspecified Pneumonia
	143	Disease of Pleura
**CMG 2009**	136	Bacterial Pneumonia
	138	Viral/Unspecified Pneumonia
	143	Disease of Pleura
**Congestive Heart Failure (Age** ≥ **45)**		
**CMG 2008**	196	Heart Failure without Cardiac Catheter
**CMG 2009**	196	Heart Failure without Cardiac Catheter
**Diabetes (All ages)**		
**CMG 2008**	437	Diabetes
**CMG 2009**	437	Diabetes
**Cardiac CMGs (Age ≥ 40)**		
**CMG 2008**	202	Arrhythmia without Cardiac Catheter
	204	Unstable Angina/Atherosclerotic Heart Disease without Cardiac Cath
	208	Angina (except Unstable)/Chest Pain without Cardiac Catheter
**CMG 2009**	202	Arrhythmia without Cardiac Catheter
	204	Unstable Angina/Atherosclerotic Heart Disease without Cardiac Cath
	208	Angina (except Unstable)/Chest Pain without Cardiac Catheter
**Gastrointestinal CMGs (All ages)**		
**CMG 2008**	231	Minor Upper Gastrointestinal Intervention
	248	Severe Enteritis
	251	Complicated Ulcer
	253	Inflammatory Bowel Disease
	254	Gastrointestinal Hemorrhage
	255	C
	256	Esophagitis/Gastritis/Miscellaneous Digestive Disease
	257	Symptom/Sign of Digestive System

## Abbreviations

ACSCs: Ambulatory care sensitive conditions
US: United States
FHO: Family Health Organization
COPD: Chronic obstructive pulmonary disease
CMG: Case Mix Group
DAD: Discharge Abstract Database
Registered Patient Database: RPDB
RIO: Rurality Index of Ontario
RUBs: Resource Utilization Bands
OHIP: Ontario Health Insurance Plan
